# Precision medicine by designer interference peptides: applications in oncology and molecular therapeutics

**DOI:** 10.1038/s41388-019-1056-3

**Published:** 2019-10-21

**Authors:** Anabel Sorolla, Edina Wang, Emily Golden, Ciara Duffy, Sónia T. Henriques, Andrew D. Redfern, Pilar Blancafort

**Affiliations:** 1 Harry Perkins Institute of Medical Research, QEII Medical Centre and Centre for Medical Research, The University of Western Australia, Nedlands, WA 6009 Australia; 2 School of Biomedical Sciences, Faculty of Health, Institute of Health & Biomedical Innovation, Queensland University of Technology, Translational Research Institute, Brisbane, QLD 4102 Australia; 3 School of Medicine, The University of Western Australia, Crawley, WA 6009 Australia

**Keywords:** Biotechnology, Cancer

## Abstract

In molecular cancer therapeutics only 10% of known cancer gene products are targetable with current pharmacological agents. Major oncogenic drivers, such as MYC and KRAS proteins are frequently highly overexpressed or mutated in multiple human malignancies. However, despite their key role in oncogenesis, these proteins are hard to target with traditional small molecule drugs due to their large, featureless protein interfaces and lack of deep pockets. In addition, they are inaccessible to large biologicals, which are unable to cross cell membranes. Designer interference peptides (iPeps) represent emerging pharmacological agents created to block selective interactions between protein partners that are difficult to target with conventional small molecule chemicals or with large biologicals. iPeps have demonstrated successful inhibition of multiple oncogenic drivers with some now entering clinical settings. However, the clinical translation of iPeps has been hampered by certain intrinsic limitations including intracellular localization, targeting tissue specificity and pharmacological potency. Herein, we outline recent advances for the selective inhibition of major cancer oncoproteins via iPep approaches and discuss the development of multimodal peptides to overcome limitations of the first generations of iPeps. Since many protein–protein interfaces are cell-type specific, this approach opens the door to novel programmable, precision medicine tools in cancer research and treatment for selective manipulation and reprogramming of the cancer cell oncoproteome.

## Introduction

Intracellular protein–protein interactions govern many facets of cellular biology and physiology, such as transcription factor (TF) binding to promoters and enhancers, localization of protein complexes in the cell and specificity of signal transduction information. Recent comprehensive analyses across cell types and cancer types have outlined a vast network of protein–protein associations [1]. Key protein–protein contacts often act as orchestrators of molecular function and are capable of remodeling the protein interaction network, leading to significant switches in cellular behavior. For example, the self-renewal network in embryonic stem cells is comprised of multiple TFs; however, ectopic delivery of only a few TFs to somatic cells (OCT4, SOX2, MYC, and KLF-4) can restore pluripotency [168*].

Not surprisingly, protein–protein networks central to oncogenesis and disease progression are highly altered during cancer pathogenesis. However, inhibiting these interactions represents a very significant challenge. Contact surfaces responsible for protein–protein interactions are relatively large (~1500–3000 Å) and grooves or binding pockets minimal or absent [2]. Unlike targetable cellular entities such as enzymes or tyrosine kinase receptors, there are no natural ligands binding to such protein interfaces [3], which limits the usual topological starting point employed for discovery pipelines of new small molecule binders. Consequently, the development of small molecule inhibitors able to bind such featureless protein interfaces is extremely difficult.

Mainly, two types of therapeutic agents are currently exploited in clinical oncology: small molecules drugs (chemicals, molecular weight (Mw) < 500 Da) and protein-based therapeutics (also referred to as biologicals; Mw > 5000 Da). Approximately, 80% of human disease targets, including those involved in intracellular protein–protein interactions, are beyond the reach of these treatment modalities [4]. Small molecule drugs can easily cross cell membranes and reach intracellular proteins, and have been successfully developed as targeted chemotherapy to inhibit oncoproteins; for example, Imatinib, a kinase inhibitor that binds to the ATP-binding pocket of BCR-ABL kinase, is used for the treatment of patients with chronic myeloid leukemia [182*]. However, single mutations on the target site can render these drugs inactive and patients rapidly acquire resistance. Due to their size, small molecule chemicals cannot target and inhibit large interface surfaces involved in protein–protein interactions. Also, as only 3000 out of ~25,000 proteins encoded by the human genome possess hydrophobic pockets, only a small fraction of potential cancer targets can be inhibited by small molecule chemicals. Additionally, more than 90% of human proteins are intracellular or secreted, which makes them difficult to bind by therapeutics that largely target cell surface proteins [5]. Intracellular proteins are inaccessible to biologicals, which are able to target larger surfaces with high specificity and successful at inhibiting cell surface proteins, but unable to enter inside cells.

**Fig. 1:**
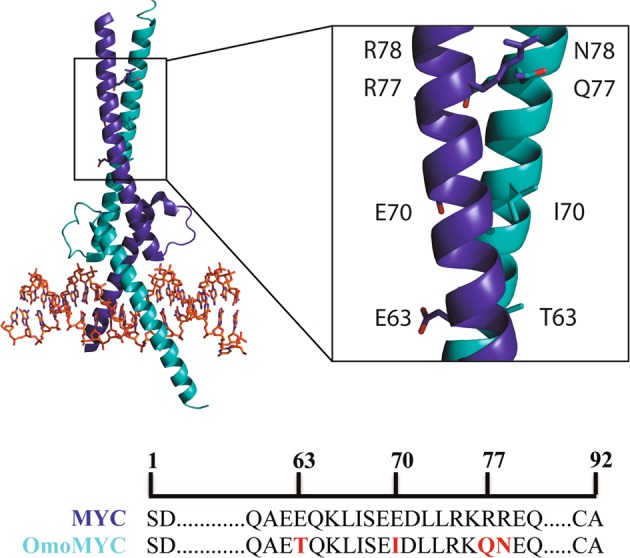
Structural representation of MYC interacting with OmoMYC. OmoMYC comprises 92 amino acids derived from MYC but differ in four amino acids positions mapping in the bHLHZip region (red boxes). Image was constructed by superposition of the crystal structures of the OmoMYC homodimer (5I50) and the MYC:MAX heterodimer structure (1NKP). Only one OmoMYC monomer and MYC monomer are shown for clarity

Inhibitory or interference peptides (iPeps) have emerged as a promising approach for the design of drug candidates. Peptides (Mw 500–5000 Da) combine the advantages of chemicals and biologicals: they can target large surface areas and therefore achieve the high selectivity of biologicals, as well as being able to enter cells and reach intracellular targets as effectively as small molecule drugs. iPeps are derived from sequences of native proteins mediating protein-protein interactions, usually comprising a small number of key residues. Such peptides act as dominant-negative versions of the endogenous proteins, binding without functionality and preventing the access and action of native proteins. Thus, by binding different partners or co-factors involved in cancer development, iPeps block the function of the endogenous proteins (Fig. 1) and impair cancer progression.

In this work, we review advances in the preclinical and clinical development of iPeps against a range of difficult-to-drug oncotargets (summarized in Table 1). We have focused on iPeps designed to inhibit the oncogenic TFs, and the proteins KRAS, BCL2, and HDM2 involved in intracellular signaling. In addition, we discuss several limitations encountered in the use of iPeps including variable cellular and nuclear penetrability, poor stability in the circulation and limited bioactivity. Lastly, we outline strategies to overcome these limitations, which include the use of unnatural amino acids, conjugation with cell penetrating sequences, cyclization, and utilization of targeted nanoparticles. In summary, iPeps are capable of a high degree of chemical versatility against a broad range of targets and thus hold enormous promise at inhibiting recalcitrant cancer targets.

**Table 1:** Examples of interference peptides, peptidomimetics, and small inhibitors utilized in preclinical and clinical studies

Target/s	Name/s	Cancer model/s	In vitro	In vivo	Concentration/dose administered	In vivo mode of administration	Clinical trials	References
MYC	Int-H1-S6A,F8A	Breast cancer cell line MCF-7	✓	×	0–10 µM	N/A	No	[15]
	Pen-ELP-H1	Breast cancer cell line MCF-7	✓	×	0–18 µM	N/A	No	[80]
	CPP-ELP-H1	Rat glioma cell C6 allograft	✓	✓	200 mg/kg	Intravenously	No	[17]
	IIA4B20 IIA6B17	Myc-transformed chicken embryo fibroblasts	✓	×	0–75 µM and 0–125 µM	N/A	No	[108*]
	10058-F4	Myc-transduced rat Rat1a xenograft in mice	✓	✓	64 µM	Cells treated in vitro and then inoculated in mice	No	[181*]
		Human prostate PC-3 and DU145 xenografts	✓	✓	20 and 30 mg/kg	Intravenously	No	[128*]
	10074-G5	Human Daudi Burkitt’s lymphoma xenografts	✓	✓	20 mg/kg	Intravenously	No	[115*]
	3jc48–3	human promyelocytic leukemia and Daudi Burkitt’s lymphoma cell lines	✓	×	0–50 µM	N/A	No	[113*]
	KJ-Pyr-9	Human triple negative breast cancer MDA-MB-231 xenograft	✓	✓	10 mg/kg	Intraperitoneally	No	[130*]
	Mycro3	Human pancreatic ductal adenocarcinoma Panc1 and MiaPaCa2 xenografts	✓	✓	100 mg/kg	Orally	No	[166*]
	OmoMYC	Human non-small cell lung cancer A549 xenografts	✓	✓	5 × 10^10^ viral particles	Intratumoral	No	[23]
		OmoMYC transgenic mice developing skin papilloma	✓	✓	N/A	expression directed to suprabasal keratinocytes	No	[19]
						under the control of the human involucrin promoter		
		Spontaneous multifocal invasive astrocytoma and human glioblastoma xenograft	✓	✓	N/A	TRE-Omomyc; CMVrtTA mouse model, in which Omomyc	No	[105*]
						Is widely expressed upon doxycycline administration		
		KRas^LSL-G12D/+^-induced lung adenocarcinoma	✓	✓	2.37 and 60/120 mg/kg	Intranasally	No	[25]
		Human non-small cell lung cancer H1975 xenograft	✓	✓	60 and 120 mg/kg	Intravenously	No	[25]
	FPPa-OmoMYC	Murine triple negative breast cancer T11 allograft	✓	✓	32.2 mg/kg	Intratumoral	No	[26]
HOX(1–8)	HXR9	Murine melanoma B16 allograft	✓	✓	10 mg/kg	Intravenously	No	[32]
		Human non-small cell lung cancer A549 xenografts	✓	✓	100 mg/kg	Intraperitoneally	No	[33]
		Human ovarian cancer SK-OV3 xenografts	✓	✓	100 mg/kg	Intravenously	No	[34]
		Human triple negative breast cancer MDA-MB-231 xenografts	✓	✓	100 mg/kg	Intratumorally	No	[37]
		Human prostate cancer LNCaP xenografts	✓	✓	100 mg/kg	Intratumorally	No	[38]
		Human Mesothelioma MSTO-211H xenografts	✓	✓	25 mg/kg	Intraperitoneally	No	[39]
		Human oral squamous cell carcinoma cells	✓	×	0–100 µM	N/A	No	[153*]
PBX1	EN1-iPep	Human and murine basal-like breast cancer cell lines	✓	×	0–100 µM	N/A	No	[45]
	EN1_act_-iPep	Murine triple negative breast cancer T11 allograft	✓	✓	25 mg/kg	Intratumorally	No	[49]
	EN1_act_-RGD_1_	Murine triple negative breast cancer T11 allograft	✓	✓	25 mg/kg	Intravenously	No	[50]
KRAS	Peptide 49 Peptide 54	Human lung cancer cell line H441	✓	×	0−0.1 mM	N/A	No	[56]
	SAH-SOS1_*A*_	Human pancreatic, colon and lung cancer cells bearing KRAS mutations	✓	×	0.625–40 µM	N/A	No	[57]
BCL2	ABT-737	Human small cell lung cancer H146 and H1963 xenografts	✓	✓	25, 50, 75, 100 mg/kg	Intraperitoneally	No	[64]
	SAHB_A_	Human leukemia xenografts	✓	✓	10 mg/kg	Intravenously	No	[63]
	ABT-263	Human B-cell lymphoma, multiple myeloma and small cell lung cancer xenografts	✓	✓	100 mg/kg	Orally	Yes	[65]
	ABT-199	Human hematological tumor xenografts	✓	✓	100 mg/kg	Orally	Yes	[66]
HDM2	^D^PMIα	Human glioma U87 xenograft	✓	✓	3, 4, 7.5, 10 mg/kg	Intravenously	No	[70]
HDM2 HDMX	SAH-p53–8	Human choriocarcinoma JEG-3 xenograft	✓	✓	10 mg/kg	Intravenously	No	[72]
	ATSP-7041	Human osteosarcoma SJSA-1 and breast cancer MCF-7 xenografts	✓	✓	15, 20, 30 mg/kg	Intravenously	No	[73]
	MCo-PMI	Human colon cancer HCT116 xenograft	✓	✓	40 mg/kg	Intravenously	No	[189*]
	ALRN-6924	Human acute myeloid leukemia xenografts	✓	✓	20 mg/kg	Intravenously	Yes	[74]

The name of the peptides, peptidomimetics, and small inhibitors, the cancer model, the application in vitro and in vivo, the concentration/dose administered, the in vivo mode of administration, the clinical trials designed and the related references are indicated. *N/A* not applicable; ✓ Yes; × Not. The references marked with an asterix can be found in Supplementary Information

### Inhibiting oncogenic TFs

#### MYC (MYC proto-oncogene, BHLH TF)

The MYC oncogene family comprises three members: c-Myc, N-Myc, and L-Myc, which have similar function but differ in potency and patterns of expression [6], [106*, 109*, 112*, 148*, 149*, 160*, 167*]. c-Myc (herein abbreviated as MYC) is a master regulator of gene transcription, controlling the expression of ~30% of genes in the human genome [118*]. MYC orchestrates a wide range of essential cellular processes, such as cell growth, apoptosis, metabolism, RNA biogenesis, and splicing [7], [107*, 119*, 126*, 142*, 161*]. Oncogenic deregulation of MYC is observed in the vast majority (~70%) of human malignancies including breast, colon, cervix, lung, bone, brain, and blood cancers [8], [101*, 120*, 141*, 163*, 175*, 178*], globally accounting for one-seventh of all cancer deaths [118*]. Furthermore, MYC is overexpressed in approximately 50% of triple-negative breast cancers (TNBCs) [127*], one of the most aggressive subtypes of breast cancer.

MYC is a TF composed of a basic helix–loop–helix leucine zipper (bHLHZip) DNA-binding domain [110*]. It does not homodimerize [154*] but forms heteromeric complexes with its TF partner MAX, among other cofactors, to bind DNA. MYC activates transcription via the association of the DNA-binding domain with a *C*-terminal activator domain [9].

Due to its nuclear localization and featureless protein structure, MYC is not an easily “druggable” protein [10], [135*]. Multiple agents have been utilized to inhibit MYC binding to cofactors or downstream effectors, such as G-quadruplex regulatory stabilizers, small interfering RNA, and small molecule inhibitors [11], [150*, 121*, 151*, 171*, 173*, 180*]. However, due to insufficient specificity, poor tumor site penetration, poor bioavailability, fast metabolism, and consequent inadequate therapeutic efficacy, the clinical application of these MYC inhibitors has been hampered [115*].

The safety of inhibiting a target responsible for such widespread control of essential cellular processes must be carefully considered. Full myc-knockout animal models demonstrate embryonic lethality [12]. However, as all studies to date have focused on the developmental role of the protein, the significance of MYC inhibition in adult cellular physiology is less established, and so the consequences of MYC inhibition for normal tissues during cancer therapy are hard to predict. In contrast to malignancy, in normal tissues MYC is tightly controlled and generally expressed at low levels except in proliferating tissues. A physiological dose response to cellular MYC concentrations is also seen in animal models generated to express MYC at a range of levels where different degrees of MYC reduction have different impacts [13]. Consequently, partial MYC inhibition could impact tumorigenesis without substantial morbidity but thorough animal toxicology work and well-considered dose escalation in early phase trials are still required.

Peptide-based inhibitors able to block protein–protein interactions not only have demonstrated high selectivity and affinity for target sites, but also low toxicity [14], features that have been harnessed to target MYC. The first attempt to inhibit MYC function utilizing an interference peptide was done by Giorello et al. using a 14 amino acid peptide designated as “H1” derived from the helix 1 *C*-terminal region of MYC [15]. The peptide was fused to penetratin to facilitate the tumor cell penetrability and delivered cytotoxic activity against MCF7 cells. The H1 peptide was also administered in vivo after docetaxel treatment to facilitate nuclear accessibility and showed tumor growth reduction as well as increased survival in mice bearing HeLa xenografts [16]. The H1 peptide has also been fused to the cell penetrating peptide (CPP) Bac, a basic peptide derived from bovine neutrophils, and to an elastin-like polypeptide (ELP), which allowed the control release of H1 after induction of local hyperthermia in a rat C6 glioma [17]. The resulting peptide fusion reduced in vivo tumor growth when administered intravenously at 200 mg/kg.

Currently, the best MYC inhibitory protein characterized that exhibits strong in vivo antitumoral activity is OmoMYC [18]. OmoMYC is a 92-amino acid protein derived from the MYC bHLHZip region having four point mutations which abolish MYC molecular recognition [18] (Fig. 1). OmoMYC has the ability to bind to MAX directly, but also forms homodimers, OmoMYC–OmoMYC, which compete with MYC-MAX heterodimers to bind DNA, interfering with the normal function of MYC in activating transcription of downstream oncogenic pathways [19–21], [157*, 164*, 165*]. Soucek and colleagues have shown that OmoMYC exhibits proapoptotic activity in myoblasts abundantly expressing MYC, but not in cells expressing very low levels of MYC [22], suggesting that MYC expression levels influence OmoMYC apoptotic activity. Systemic inhibition of MYC by OmoMYC in KRAS-driven lung cancer [23], in MYC-induced papillomatosis [19] and in glioma [24] was also shown to have a profound therapeutic effects, and yet only elicited mild and rapidly reversible side effects on normal tissues. These minimal side effects demonstrate the safety and potential applicability of OmoMYC which should contribute to its success in patients [20, 21]. Very recently, OmoMYC was able to abrogate the growth of lung adenocarcinomas in mice when administered intranasally or systemically [25]. These preclinical successes should lead to the entry of OmoMYC into clinical phases of development in the near future.

We have shown that OmoMYC is able to inhibit the growth of breast carcinoma cells when injected orthotopically in TNBC animal models having intact immune systems [26]. In addition to potent tumor inhibition and induction of Caspase-3 apoptosis, OmoMYC induced a suppression of the immune checkpoint protein PD-L1. Moreover, we found that penetrability of the OmoMYC peptide was a limiting factor for its therapeutic activity in these cancers [26]. To overcome this, we engineered the OmoMYC sequence with state-of-the-art cell penetrating sequences selected from large peptide libraries (Phylomers). One such fusion inhibited multiple MYC-dependent signaling pathways in TNBC cells involved in cell growth, RNA processing and metabolism, and as consequence strongly inhibited the growth of TNBC allografts [26].

Peptidomimetics, including non-peptidic compounds designed to mimic the binding of a peptide sequence to a target, are attractive therapeutic agents given their enhanced proteolytic stability over the native peptide sequence and their potential to be orally bioavailable [183*]. Several small molecule peptidomimetics able to disrupt protein–protein interactions of MYC with its binding partners and DNA have also been reported. The peptidomimetics II6B17 and IIA4B20 [184*] inhibit MYC/MAX dimerization and MYC-induced transformation of chicken embryo fibroblasts in in vitro assays [108*]. In addition to OmoMYC, other MYC interference peptides, MYC peptidomimetics and small molecules capable of inhibiting MYC are summarized in Table 1.

#### HOX (Homeobox) TFs

In mice and humans, *HOX* genes dictate body patterning and segmentation during development [27]. *HOX* genes encode for 38 different homeobox-containing TFs grouped into four genomic clusters, *HOXA* to *HOXD* [143*, 158*].

In spite of the highly specific in vivo biological functions of the HOX TFs, these proteins bind with relatively low affinity to DNA [28], requiring a cofactor to increase their affinity and specificity. Indeed, the formation of a cooperative DNA binding complex including HOX proteins and the cofactor Pre-B-cell Leukemia Homeobox (PBX) significantly increases the affinity and specificity of HOX proteins for DNA [29].

The importance of HOX proteins in malignancy first became apparent through observing their involvement in oncogenic gene fusion events for haemopoietic malignancies [30]. In addition, dysregulation of HOX proteins in cancer is relatively common, although complex, with different family members showing altered expression in different tumor types (reviewed in ref. [31]). *HOX* genes are frequently overexpressed in hematologic malignancies [122*] and solid tumors [31–34], [102*, 114*, 116*, 133*, 137*, 140*, 145*, 179*]. Direct involvement in cancer pathogenesis is likely with roles established for HOX-family members in proliferation, angiogenesis, and metastasis [32], [132*, 136*]. However, examples of suppressive influences on tumor progression also exist, for example by HOXA5 in the maintenance of the epithelial phenotype, and HOXA4 in the inhibition of tumor cell migration [170*]. Thus, HOX-based treatment would need be finessed with different family members being targeted to treat particular cancers.

Considering toxicity, some functionalities in normal adult tissues have been defined for the group such as the maintenance of adult haematopoietic stem cells by HOXA proteins [139*], and the control of endometrial receptivity by HOXA and HOXD proteins [169*]. While potential toxicities require consideration in clinical development the governed processes in adults appear relatively limited such that toxicities should be manageable.

Interestingly, in contrast to pro-malignant roles that tend to involve HOX:PBX interactions in gene control, tumor suppressor roles often involve HOX proteins interacting alone with DNA such as in E-cadherin regulation [170*]. Consequently, targeting the HOX:PBX heterodimer, such as by HXR9 as discussed below, may yield more selective therapeutic effects over HOX-targeting alone [147*].

Papadopoulos et al. demonstrated that the ectopic expression of the C terminus Scr gene containing a Hox binding site and the YPWM motif causes changes in tissue fate in *Drosophila* [152*]. Also, synthetic HOX hexapeptide motifs, peptides containing a conserved motif of six amino acids from the native sequence of HOX proteins, have been shown to compete in vitro with the HOX–PBX1complex, disrupting cooperative DNA binding [35]. Morgan et al. demonstrated that the cell permeable peptide HXR9 binds to PBX (which interacts with HOX proteins comprising 1–8 paralogues) and disrupts the binding of HOX/PBX dimers to the DNA in melanoma cells resulting in apoptosis induction [32]. HXR9 is an 18 amino acid peptide [147*] consisting of a duplicate of the Hox protein hexapeptide motif that interacts with PBX proteins to confer the interfering function, plus a CPP at the C terminus [36]. The CPP has nine arginine residues, R9, which facilitates cell entry [32]. In vivo, the intravenous administration of HXR9 inhibited growth in the B16 melanoma animal model. The same peptide induced cytotoxicity in the renal cancer cells CaKi-2 and 769-P [36]. In addition, HXR9 triggered apoptotic cell death in the non-small-cell lung cancer cells A549 and H23 and reduced in vivo tumor growth of A549 xenografts when administered into the peritoneum or in the tumor [33]. HXR9 also caused apoptosis in the ovarian cancer cell line SK-OV3 and inhibited tumor growth of SK-OV3 xenografts [34]. Moreover, HXR9 induced apoptosis in breast cancer cell lines, correlating with their higher expression levels of *HOX* genes, and HXR9 injected intratumorally retarded in vivo tumor growth in MDA-MB-231 TNBC xenografts [37]. Furthermore, HXR9 had apoptotic activity in prostate cancer cells and inhibited in vivo tumor growth of LNCaP xenografts [38]. Finally, HXR9 drove apoptosis in all malignant mesothelioma cell lines tested in Morgan’s study, which correlated with their *HOX* genes expression levels. The peptide also reduced in vivo tumor growth of MSTO-211H xenografts when injected intraperitoneally [39].

Considering potential toxicities of HXR9, no significant toxicities have been apparent in studies to date although adverse effects are frequently not discussed specifically. For example, HXR9-treated animals showed no weight loss relative to control animals [185*]. Another study noted lack of toxicity in mice regarding blood counts and liver histology changes [32]. However, no toxicity reports were made for mice bearing xenografts from ovarian [34], mesothelioma [39], prostate cancer [38], meningioma [186*], melanoma [187*], or breast cancer [37].

A second-generation derivative of HXR9, HTL-001 (HOX Therapeutics Ltd.), has now undergone animal safety testing with no substantial toxicities identified. Activity in glioblastoma xenograft models was recently demonstrated for HTL-001 [40]. Commencement of an early phase human clinical trial in glioblastoma with HTL-001 is planned for the second half of 2019 (https://www.hoxtherapeutics.com/our-technology/development-pipeline).

#### Engrailed TFs

Another important group of players participating in cooperative DNA binding with PBX are the gene products of the *ENGRAILED* (*EN*) genes. *EN* genes were initially discovered as essential genes for embryonic development in arthropods [41] and later for the patterning of the midbrain–hindbrain boundary region of the central nervous system in vertebrates. Knockout mice for En1 and En2 develop dopaminergic neuronal degeneration by caspase-3-mediated apoptosis [42], [103*, 159*]. En1 loss-of-function mutations have been linked to progressive cell degeneration leading to onset of Parkinson’s disease [43] and EN2 has been associated with autism in genetic linkage studies in humans [44]. Orthotopic infusion of En1 and En2 proteins protect dopaminergic neurons from neurotoxins in the mouse midbrain [42].

**Fig. 2:**
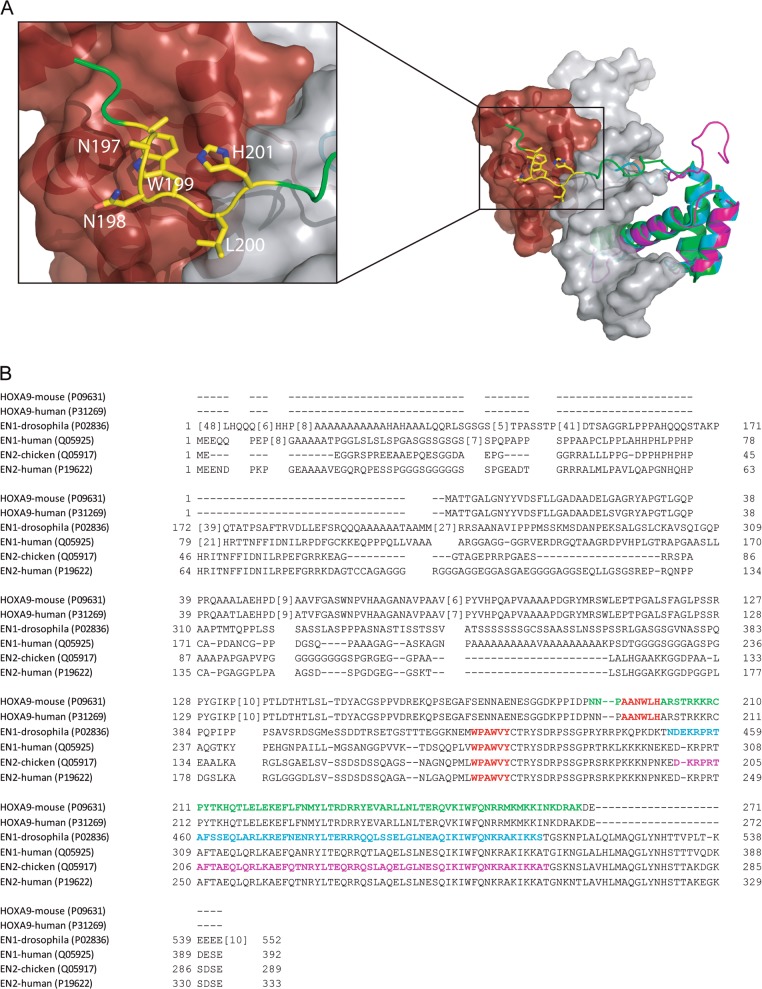
Structural representation of HOXA9 interacting with PBX1 and the DNA. **a** Crystal structure (1PUF) of the homeodomain of HOXA9 (green) interacting with PBX1 (maroon surface representation) and DNA (gray surface representation). The inset shows the interaction of the hexapeptide motif of HOXA9 (yellow sticks) with the binding pocket of PBX1. Crystal structures of the homeodomain of EN1 (2JWT, cyan) and EN2 (3ZOB, magenta) are shown superimposed on HOXA9. **b** Alignment of protein sequences of EN1, EN2, and HOX. Red and bolded text indicates the hexapeptide motifs of each protein

Engrailed proteins, as well as HOX proteins and PBX proteins, contain a ~60 amino acid homeodomain. Also conserved in EN proteins are four EN-specific domains, namely EH1, EH2, EH3, and EH5, with EH4 encoding the homeodomain. The EH2 domain contains a PBX-interaction motif necessary for cooperative DNA binding with PBX [35] (sequence: WPAWVY), in which the two tryptophan residues are essential for such interaction and binding. Similarly, HOX proteins present a hexapeptide sequence with two highly conserved tryptophan residues essential for cooperative DNA binding with PBX (Fig. 2b).

In addition to the fundamental role of EN1 proteins during embryonic development, mounting evidence points to a role for these factors as oncogenic drivers. Our group first reported that *EN1* is selectively overexpressed in basal-like breast cancers [45]. Later, Kim et al. demonstrated that *EN1* is overexpressed in quintuple negative breast cancers and its presence is associated with reduced overall survival in patients [138*]. The excretion of EN2 in the urine has been found to be a useful biomarker for bladder and prostate cancer [46], [146*]. Thus, selectively blocking the action of these TFs is of great interest in oncology, given their implication in the progression of a wide range of human cancers.

Dynamic simulations have confirmed that EN1-iPeps acquire helical conformation and mimic the relative motif in full EN1 protein, suggesting functional interference may be achieved [47]. Beltran et al. [45] designed different peptides derived from the human EN1 gene and demonstrated that, in basal-like breast cancer cells, these peptides were able to bind PBX1 and PAX6, disrupting cooperative DNA binding. The peptides mimic the highly conserved hexapeptide motif present in the EN1 [47] (Fig. 2b). To facilitate internalization, a nuclear localization signal (NLS) sequence derived from the Simian Virus 40 (SV40) large T antigen with cell penetrating properties was added at the *N*-terminus of the sequence [48]. Several peptide variants were tested in this study (EN1-iPeps) which were capable of selectively reducing cell survival of basal-like breast cancer cell lines (EN1^+^) without affecting EN1^−^ cells [45]. Going beyond the single hit approach of TF inhibition alone, Sorolla et al. recently reported docetaxel sensitization in vivo in TNBC employing docetaxel nanoparticles encapsulating EN1-iPeps [49]. Later, EN1-iPeps engineered with an RGD cell localizing component were co-delivered with docetaxel using nanoparticles resulting in in vivo TNBC growth inhibition [50]. At this stage we are not aware of any EN1-iPep yet at the trial-ready stage although considerable optimization work is likely to be ongoing.

### Inhibiting intracellular oncoproteins involved in signaling pathways

A myriad of intracellular proteins involved in cancer progression, such as the central oncoproteins KRAS, BCL-2, and HDM2, remain difficult to target pharmacologically. Intensive research has been done to suppress these cancer-associated pathways by targeting and inhibiting protein–protein interfaces via iPep approaches.

#### KRAS

KRAS and the other RAS isoforms, NRAS and HRAS, are small GTPases involved in pathways important for cell growth, differentiation and survival. RAS proteins are mutated in 30% of all malignancies and are regarded as important anticancer targets. The KRAS isoform is particularly relevant in pancreatic cancer as it is mutated in 90% of the patients [51]. Activating mutations render RAS constitutively active, which in turn activates RAF and the downstream survival pathways PI3K and MAPK, inducing cell growth, proliferation, protein synthesis, and apoptosis inhibition. Despite their potential as therapeutic targets, RAS isoforms are difficult to target. Small molecule drug inhibitors that compete with ATP for its binding pocket on kinases are successful at blocking kinase activity, but inhibitors that target GTP binding pockets in RAS have proven ineffective due to the high-GTP affinity of RAS. Several drugs with inhibitory activity for KRAS have been tested preclinically, however, until recently, none had demonstrated significant therapeutic effect in animals for subsequent clinical development in humans [52]. Recent promise has emerged through the use of covalent G12C-specific small molecule inhibitors targeting the mutated cysteine associated with the switch II pocket adjacent to the GTP-binding site. This small molecule inhibited RAS and cell viability at the nanomolar range of concentrations in KRAS^G12C^-mutant cell lines [53], [124*]. This work has culminated in a phase I trial of lead compound AMG 510 (NCT03600883) which produced partial responses in 50% of evaluable patients with KRAS G12C-mutant non–small cell lung cancer, and stable disease in the majority of patients with colorectal or appendiceal cancer [54]. This trial is ongoing with the duration of observed responses yet to be determined.

In the past few years there has been mounting interest in the development of iPeps targeting KRAS. Destabilizing the interactions between KRAS and its binding partners SOS1 or Raf, are two potential strategies to inhibit KRAS activity. Cyclic peptides identified from screening a peptide library against the oncoprotein KRAS^G12V^, the most common mutation in KRAS, have demonstrated specific binding to KRAS and inhibition of the Raf-KRAS interaction at nanomolar concentrations [55]. However, these peptides lacked antiproliferative activity in cell lines in vitro, presumably due to lack of cell penetration. Interestingly, Trinh et al. identified cyclic peptides, also through screening of a peptide library against the oncoprotein KRAS^G12V^, able to internalize inside cells and induce apoptosis in vitro, but these effects were only achieved at very high concentrations of peptide [56]. Notably, Leshchiner et al. designed peptides to recapitulate the secondary structure of the KRAS-interacting α-helix of SOS1. The resulting peptides were able to bind to wild type and all mutant forms of KRAS with high affinity (nanomolar range) and displayed similar antitumoral activity in vitro in both KRAS wild type and in mutant cell lines, thus generating concerns about the tumor selectivity of the peptides [57].

Suppression of RAS dimerization represents another interesting approach for inhibiting RAS activity. Consequently, efforts have been made to identify molecules able to interact with the α4–β6–α5 region/interface of RAS to disrupt dimerization. This interface was a previously unknown region able to inhibit RAS function [58]. Monobodies, small proteins with some antibody-like features, have been successfully designed to bind to particular protein domains. The monobody NS1, designed for inhibiting RAS dimerization, effectively blocked CRAF–BRAF heterodimerization and activation [58], and more recently, has been shown to inhibit Ras-driven tumor growth in mice [59].

However, in vivo toxicity of inhibitory peptides designed against KRAS remains largely unassessed. Only Leshchiner et al. reported lack of in vivo toxicity of SAH-SOS1_A_ in their *Drosophila melanogaster* model [57]. Thus, extensive toxicology assessments need to be performed to validate therapeutic potential in humans.

#### BCL-2 (B-cell lymphoma 2)

Proteins of the B-cell lymphoma 2 (BCL-2) family control the intrinsic pathway of apoptosis. They form homodimers and heterodimers through BCL-2 homology (BH) domains inclining cellular balance either to proapoptotic or antiapoptotic processes [60]. Since a hallmark of cancer is the evasion of apoptosis [129*], it is not surprising to find overexpression of antiapoptotic proteins and under-expression of proapoptotic proteins in many human malignancies. For example, the anti-apoptotic member BCL-2 is overexpressed in 90% of follicular B-cell lymphomas [61] while the proapoptotic member PUMA is deleted in a range of cancers [62].

The BH3 domain of BCL-2 members, which is essential for the folding integrity of a hydrophobic pocket with which BLC-2 proteins interact, is a key domain for the regulation of apoptosis. BCL-2 and BCL-X_L_ bind the proapoptotic proteins BAK and BAD utilizing this domain, resulting in apoptosis inhibition. BH3 domain-derived iPeps have been synthetized to activate apoptosis and induce tumor regression. Early work in this area showed promise with stapled peptides (see section 4.2) such as stabilized alpha-helix of BCL-2 domains (SAHBD) showing the ability to inhibit the growth of leukemia xenografts in mice [63].

BH3 peptidomimetics designed to mimic the binding of the BH3 domain-derived peptides to BCL-2 have demonstrated significant therapeutic value as BCL-2 antagonists [156*, 188*]. The peptidomimetic ABT-737 demonstrated reduction of tumor growth in small cell lung cancer xenografts through proapoptotic mechanisms [64]. An orally bioavailable derivative of ABT-737, ABT-263 [65] (navitoclax), has now successfully navigated clinical trials for chronic lymphocytic leukemia (CLL), being shown to reduce lymphocyte counts by over 50% in 90% of patients with a progression-free survival of 25 months leading to an FDA approval for this indication in 2016. Thrombocytopenia due to BCL-X_L_ inhibition was the major dose-limiting toxicity [156*]. Subsequently, a BCL-2-specific inhibitor has been developed, ABT-199 (venetoclax) [66], to avoid potential toxicity. Preclinical work with ABT-737 revealed that this drug can induce resistance through the upregulation of other antiapoptotic proteins [67] and so there were initial concerns that the even narrower targeting spectrum of venetoclax may lead to attenuated results. However, to date activity of venetoclax has been promising across a range of hematological malignancies with a response rate of 79% in relapsed CLL and complete responses in 20%. The potency of activity was evidenced by the occurrence of three episodes of tumor lysis syndrome at higher doses (NCT01328626). More impressive still, in the randomized Phase III MURANO study, venetoclax in combination with the anti-CD20 antibody rituximab, achieved a vastly superior rate of undetectable minimal residual compared to rituximab and chemotherapy [68]. Venetoclax is now entering trials for the treatment of solid tumors, particularly breast (NCT03584009) and prostate cancer (NCT03751436) in combination with endocrine agents.

#### HDM2 (human double minute 2 homolog)

p53 is a key tumor suppressor that induces DNA repair, and/or apoptosis. p53 function is regulated by human double minute 2 (HDM2) and HDMX, two proteins that can inhibit its activity in healthy cells. Overexpression of HDM2 and HDMX occurring in some cancers dysregulates the p53-associated pathway. HDM2 is an E3 ubiquitin–protein ligase recognized as an excellent cancer target. HDM2 binds to p53 with a 15 amino acid region that folds as an α-helix flanked by highly hydrophobic interfaces [69]. The HDM2-p53 interaction inhibits the transcriptional activity of p53 and promotes rapid degradation of p53, thereby favouring tumor progression. Small molecules or short peptides able to block HDM2-p53 binding have emerged as attractive therapeutic agents for malignancies harboring wild-type p53.

Liu et al. identified the D-peptide inhibitor of p53-HDM2 interaction, ^D^PMIα. Such peptide, encapsulated in liposomes, demonstrated therapeutic activity in glioblastoma xenografts generated with U87 cells (containing wild-type p53 and overexpressing HDM2) [70]. However, ^D^PMIα did not show significant activity in p53-mutant human glioma U251 cells [70]. Harbour et al. reported αHDM2, a 12 amino acid peptide derived from the p53 sequence that mediates the binding to HDM2 fused to the cell penetrating peptide TAT, causing cell death in retinoblastoma and uveal melanoma cell lines and tumor destruction in an intraocular retinoblastoma mice model [71]. In contrast with ^D^PMIα, αHDM2 induced cell death in both wild-type and p53-mutant CC3A cells [71].

It has been suggested that inhibition of HDM2 is not fully effective in tumors overexpressing the other p53 negative regulator HDMX due to its ability to also sequester p53 [72]. Thus, there is an increasing interest in developing HDMX inhibitors and HDM2/HDMX dual inhibitors. Bernal et al. developed SAH-p53-8, a peptide with high affinity for HDMX, which showed robust tumor growth reduction in a JEG-3 choriocarcinoma xenograft model [72]. This peptide was ineffective against the p53-mutant cell line A432 and p53-mutant-induced cell lines HTC116 and SJSA-1 [72]. Chang et al. demonstrated that the stapled α-helical peptide ATSP-7041 binds and inhibits both HDM2 and HDMX. This peptide suppressed tumor growth of SJSA-1 (osteosarcoma) and MCF7 (breast) xenograft models, both overexpressing HDM2 and HDMX, in a p53-dependent manner [73]. This peptide was ineffective at inducing expression of p53-target genes and cell death in the p53-mutant cell lines SW480 and MDA-MB-435 [73]. Another cyclic polypeptide, MCo-PMI, with a design based on the naturally occurring cyclized peptides in plants, antagonized the interaction between p53 and HDM2/HDMX. This peptide was only active in cells harboring wild-type p53 HCT116, LNCaP, and JEG3 cells, and reduced tumor growth of HCT116 xenografts in vivo [189*]. Recently, ALRN-6924, a dual HDM/HDMX inhibitor developed by Aileron Therapeutics, revealed improved survival in mice bearing wild-type p53 acute myeloid leukemia xenografts [74], whereas p53-mutant leukemia cell lines HL60 and Kasumi-1 were insensitive to the peptide [74]. This inspired the design of an ongoing phase I clinical trial (NCT02909972) in patients with relapsed/refractory acute myeloid leukemia and advanced myelodysplastic syndrome characterized by wild-type p53. This agent is also under a phase II trial for relapsed T-cell lymphoma with preliminary results released in 2018 showing responses in 21% of 14 participants and clinical benefit in 36% [162*]. Similarly, ALRN-6924 is under evaluation in a phase I/II clinical trial in patients with wild-type p53 advanced solid malignancies and lymphomas (NCT02264613). Further to this, combination studies have also commenced with ALRN-6924 to be given with paclitaxel in solid tumors (NCT03725436) and with the CDK4/6 inhibitor palbociclib in MDM2-amplified tumors.

### Limitations of interference peptides

Peptides are excellent therapeutic agents, strengths being high selectivity, potency and safety [75]. Despite the exciting inhibitory activity of iPeps against intracellular cancer targets, some intrinsic chemical features such as cell penetration properties, stability in vivo and potential for immunogenicity can be optimized. Anticancer peptides can benefit from a range of chemical modifications (summarized in Table 2) to enhance their therapeutic potential facilitating clinical translation.

**Table 2:** Peptide limitations and strategies to overcome the limitations

Peptide limitation	Strategies to overcome peptide limitations	References
Poor cell membrane permeability	Cell penetrating peptides	[15–17, 26, 45, 48–50, 56, 71, 77, 79, 80, 100], [177*]
Poor nuclear localization	Nuclear localization sequences	[45, 48–50]
Metabolic instability and short half-life in circulation	d-amino acid substitution	[32, 36, 82]
	Un-natural amino acid substitution	[84, 85], [172*]
	Cyclization	[57, 86, 87]
	Pegylation	[111*]
	XTEN conjugation	[89, 90], [104*]
	Encapsulation with nanoparticles	[49, 50, 93, 95–98]
Rapid clearance	Linkage to AG10	[91]
Poor activity	Encapsulation with nanoparticles	[94–96]
Immunogenicity	d-amino acid substitution	[190*]
	Retro-inverso d-peptides	[190*]
	Pegylation	[6, 111*, 191*]

The peptide limitations, the strategies to overcome the limitations and the related references are indicated. The references marked with * can be found in Supplementary Information

### Solutions to overcome limitations of multimodal interference peptides

#### Enhancing cellular internalization—addition of cell-penetrating peptides and nuclear localization sequences

Despite peptides being excellent therapeutic agents [75] they can exhibit limited cellular penetrability and suboptimal subcellular localization. These two limitations can be overcome by the linkage of the therapeutic peptide to a CPP and/or NLS. CPPs are peptide sequences able to cross cell membranes and deliver cargoes inside cells, mediating the entry of peptides, nanoparticles, small molecules, and nucleic acids. CPPs are usually short cationic and/or amphipathic peptides able to interact with the highly negatively charged surface proteoglycans of the cell membrane [76].

The first polycationic molecule described that facilitated cellular uptake was the trans-activating transcriptional activator (TAT) from human immunodeficiency virus 1 (HIV-1), which demonstrated cell penetrating activity in mammalian cells in tissue culture [77], [177*]. Another peptide, penetratin, was derived from the third helix of the Antennapedia homeobox peptide which was discovered to enter neuronal cells facilitating neuronal morphogenesis [134*]. Comprehensive structural and functional studies have deciphered the essential amino acids required for the cellular internalization of these proteins, which resulted in the generation of the first CPPs. The TAT cell penetrating sequence and the penetratin peptide contain 13 and 16 amino acids respectively [77, 78].

The TAT CPP successfully mediated delivery of iPep αHDM2 as mentioned previously [71]. In another study, conjugation with TAT also conferred a protective role against cellular degradation on the amphipathic α-helical anticancer peptide HPRP-A1 in HeLa cells [79]. As for penetratin, in addition to the aforementioned iPep H1 [15, 16], this CPP promoted intracellular delivery of thermosensitive ELP conjugated to H1 in MCF7 cells [80].

New methodologies based on large screens have led to the identification of novel peptides with cell penetration characteristics. Phylomer libraries, which comprise hundreds of billions of peptides present in bacteria and archaea, enabled the identification of novel functionalised penetrating peptides (FPPs) [176*]. The screening of Phylomer libraries has been coupled to high-throughput GFP complementation assays for the identification of those FPPs exhibiting superior internalization properties and prevention of endosomal trapping [144*]. Avoiding endosomal trapping is one of the most substantial limitations when delivering cargoes into the cytoplasm which most CPPs in the literature cannot yet achieve [123*]. Very recently, the FPP 1746 showed superior cellular internalization compared to TAT in HEK-293 cells [131*]. The same FPP facilitated the first successful in vivo delivery of OmoMYC in a TNBC model [26].

**Fig. 3:**
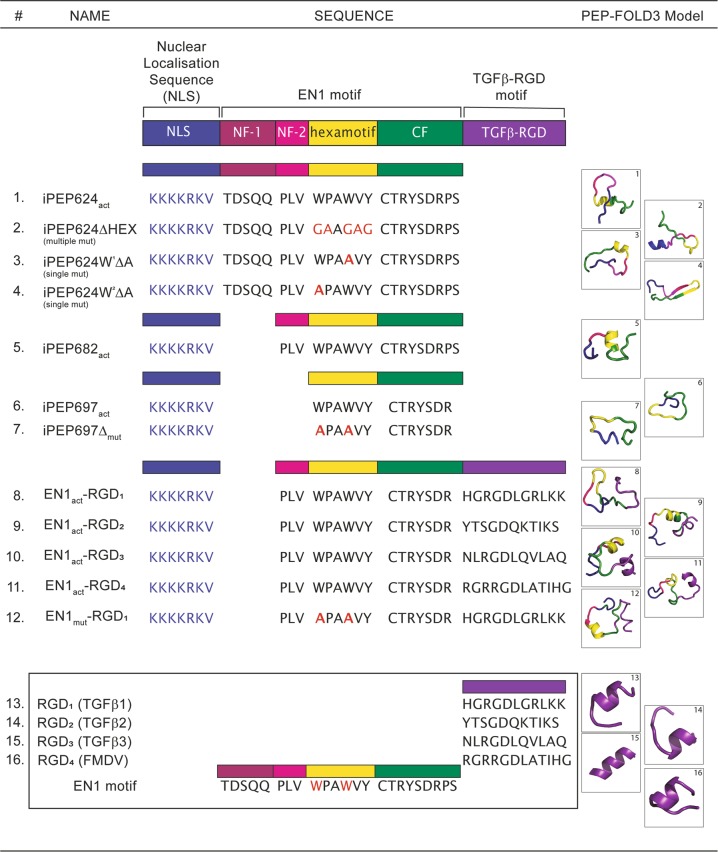
Characterization of all the interference peptides to target the EN1 TF. The table contains the name, the amino acid sequence and the tridimensional structure prediction determined by using the PEP-FOLD3 software of all the interference peptides against EN1 designed, synthetized and published. EN1 peptides were designed based on a highly conserved region of EN1 centered on the hexapeptide motif and the *N*-terminal and *C*-terminal flanking regions (NF and CF, respectively). All peptides include a nuclear localization sequence (NLS) at the *N*-terminus for targeting of the peptide. Additionally, peptides were constructed with RGD peptides at the *C*-terminus

Conjugation with an NLS is particularly important for peptides designed to target nuclear TFs. A large number of different NLS have been reported to facilitate nuclear access of several protein/peptide cargoes; For example, the NLS sequence KKKRKV from the SV40 large T-antigen, successfully mediated internalization of iPeps derived from the TF EN1 in vitro [45] and in vivo [49, 50] in TNBC models (Fig. 3). SV40 large T-antigen was also used to improve the nuclear delivery of the peptides MPG and Pep-1, which are rich in hydrophobic residues [48].

#### Enhancing the in vivo stability of peptides

One major hurdle for the clinical development of therapeutic peptides is their sensitivity to proteases which reduce their half-life upon systemic administration [81]. The most common structural modifications adopted to enhance peptide stability are chemical modifications in the *N*- and *C*-termini of the peptide backbone (e.g., acetylation of the *N*-terminus, amidation of the *C*-terminus), introduction of d-amino acids, unnatural (for example, nonproteogenic) amino acids and peptide backbone cyclization. Engineering peptides by introducing dextrorotary (d)-amino acids instead of levorotatory (l) forms is an effective strategy to avoid proteolytic degradation by proteases. d-amino acids produce conformational changes in the proteins that make them less recognizable by l-protein enzymes such as proteases. In addition, d-amino acids residues are not very common in nature, which makes them immune-inert when entering the host organism. In addition to increased half-life and lower immunogenicity, some peptides with d-amino acid residues have been shown to be more potent and penetrable into the cell [82], [125*, 172*]. Buckton et al. demonstrated that four out of five substitutions of l- for d-stereoisomers in the LB51 peptide increased the cell permeability in Caco-2 cells [82]. Veine et al. reported an increase in potency of 27,000 to 150,000-fold for the affinity of the peptide PhScN for α5β1 integrin, after the replacement of histidine and cysteine residues for their respective d-enantiomers [172*]. However, the amino acids residues to substitute in an iPep need to be carefully considered. Whilst d-amino acid substitution can increase cellular uptake of CPPs, in other cases, it can make the iPep unable to bind protein-protein interfaces when these substitutions are introduced in its active sequence.

Examination of the peptide repository PEPlife clearly shows that therapeutic peptides containing unnatural amino acids possess longer half-lives than those containing natural amino acids [83]. Many therapeutic peptides containing unnatural amino acids have been approved by the FDA such as degarelix to treat prostate cancer, semaglutide for type 2 diabetes or carbetocin, an oxytocin analog containing methyl-tyrosine, to treat postpartum hemorrhage [155*]. Similarly, a recent preclinical study from Oliva et al. reported that the introduction of cysteine derivatives and 6-aminohexanoic acid residues protected anti-microbial peptides produced from exopeptidases, increasing their potency [84]. Further, the substitution of methionine residues by norleucine and homoserine in the peptide “YSA” targeting the EphA2 receptor increased the stability in serum of the peptide and reduced in vivo prostate tumor growth and tumor vasculature in mice when conjugated with paclitaxel [85].

In addition to unnatural amino acids, peptides can be cyclized to enhance half-life [75]. This can be done by different modalities such as the establishment of disulfide bonds between two cysteines, by adding an amide bond between the *C*- and *N*-terminus of the peptide (head-to-tail cyclization), or between the side chains of natural (such as lysine and aspartic acid) and unnatural (such as ring-closing metathesis) amino acids, a process named side chain cyclization. These two latter methods are also adopted for construction of “stapled peptides”. It has been found that a number of important target protein-protein interactions for cancer drug development are mediated by peptide sequences with a α-helix structure [192*, 193*]. In addition to increasing stability against proteolysis, peptide “stapling” can help stabilize the alpha-helix conformation of an iPep, enhancing the binding of the iPep to the target protein–protein interface. Furthermore, peptide stapling has also been shown to improve cell penetrability [193*]. The stabilization of the α-helices of StAx-35R improved binding to β-catenin and disruption of β-catenin-TCF4 binding [194*]. The α-stabilized peptide SAHM1 showed successful binding to the NOTCH1-CSL complex as well as the ability to compete with the co-activator factor of the complex, MAML1. This resulted in repression of NOTCH signaling and tumor growth regression in a murine NOTCH-driven leukemia model [195*]. Other examples of stapled peptides with anti-tumoral activity in vivo include the Bcl-2 iPep SAHB_A_ [63] and the HDM2/HDMX iPeps SAH-p53-8 [72], ATSP-7041 [73], and ^D^PMIα [70] (Table 1). Peptide stapling has been exploited for the design of HDM2/HDMX iPep drug candidate ALRN-6924, which is currently undergoing clinical development [74].

Peptide cyclization mediates the stabilization of bioactive peptides such as toxins rich in cysteines [86]. An interesting application of cyclization is to keep the integrity and enhance the selectivity of linear, tumor-targeting peptides such as RGD peptides. RGD peptides or peptides containing Arg-Gly-Asp (Fig. 3) are widely used as targeting ligands for therapeutic agents given their high affinity for αvβ3, αvβ5, αvβ6, αvβ8, α5β1, and αIIbβ3 integrins overexpressed in solids tumors and associated vasculature. Cyclization of RGD peptides improves targeting affinity and selectivity [87].

#### Reducing immunogenicity

Immunogenicity of foreign peptides starts with a proteolytic process carried out by antigen presenting cells (APC). Some of the small resulting peptides will bind to MHC class II molecules and they will be then presented on the APC’s surface to be recognized by a T-cell receptor of CD4+ T cells. This will culminate in T-cell activation and antibody production [196*]. Despite the fact that peptides possess low immunogenicity compared to bigger molecules such as proteins or antibodies, they still could benefit from different strategies to reduce the probability to elicit an unwanted immunoreaction. Structural modifications such as d-amino acid residue substitution and reversal of the amino acid sequence can avoid or delay proteolytic cleavage and thus mitigate immune system recognition and immunogenicity. Both strategies have been applied to peptides used as blood brain barrier shuttles. The new peptide variants presented less immunogenicity compared to the native l-form, while maintaining activity [190*]. Pegylation is also one of the most used strategies to prevent immunogenicity of protein therapeutics. Polyethylene glycol (PEG) itself is immune-inert and in aqueous media prevents the access of proteases or peptidases to the protein/peptide by steric hindrance although it also compromises their bioactivity [197*]. Dharap et al. successfully conjugated PEG to peptide-drug conjugates composed of camptothecin and synthetic peptides similar to luteinizing hormone-releasing hormone and the BCL-2 homolog 3 peptide. The pegylated conjugates induced more apoptosis than un-pegylated camptothecin in ovarian cancer cells [191*]. Some therapeutic pegylated polypeptides have been approved by the FDA for cancer treatment such as PEG-interferon α-2b (Sylatron^TM^) [111*] for the treatment of stage III melanoma in 2011 or PEG-asparaginase (Oncaspar^®^) for acute lymphoblastic leukemia in 2005. In all these cases pegylation conferred less immunogenicity to these peptides [88].

#### Addition of biomimetic compounds

The addition of biomimetic compounds such as PEG is a common strategy for camouflaging anticancer peptides from the immune system. In addition, pegylation increases the retention time of peptides by increasing their hydrodynamic properties, which prolongs their half-life in circulation. Several peptides and proteins already approved for anticancer treatment are conjugated with PEG [111*]. However, due to the toxicity of PEG in some patients, novel biomimetic compounds presenting less immunogenicity have been developed, such as the polypeptide XTEN [89]. XTEN conjugation to teduglutide, a glucagon-like peptide-2 (GLP2) analog, resulted in an increase in the half-life of teduglutide in human plasma [90] and in rodents and monkey plasma [104*]. Other strategies reported to increase the half-life of therapeutic peptides are the fusion to unstructured proteins, to antibody Fc domains, to human serum albumin [89] and to AG10, a small molecule presenting high affinity to the plasma protein transthyretin [91].

#### Encapsulation of bioactive peptides with nanoparticles

Another strategy to protect peptides from degradation in plasma is their encapsulation in nanoparticles. This is of special interest in the development of tumor peptide vaccines where the antigens are long polypeptides and proteolytic degradation must be avoided before immunoresponse in target cells can occur [92]. Many nanoparticle formulations have been described for encapsulating tumor peptide vaccines including liposomes, polymeric micelles, polymeric nanoparticles, gold nanoparticles, nanoemulsions, and nanogels. Each nanoparticle formulation possesses advantages and disadvantages [174*]. Polymeric nanoparticles mediate excellent encapsulation of bioactive molecules and enhanced proteolytic protection compared with other nanocarriers. Luo et al. synthetized polymeric PC7A nanoparticles encapsulating the antigenic peptide OVA and observed reduction of tumor growth in melanoma, colon cancer, and human papilloma virus-E6/E7 tumor models [93].

Apart from delivering tumor peptide-based vaccines, nanoparticles enable the delivery of proapoptotic or active proteins in cancer cells [94]. Gold-based nanocapsules are stabilized through supramolecular interactions between the nanoparticle, the protein cargo and fatty acid molecules engineered in the core of the capsule. This approach has the advantage of avoiding proteolytic degradation and endosomal trapping thereby allowing the delivery of unmodified proteins. These nanoparticles showed an effective delivery of caspase-3 and induction of apoptosis in HeLa cells [95]. Similarly, proteins such as caspase-3 can be covalently linked to a thin polymer shell for systemic delivery in vivo [180*]. Bale et al. engineered hydrophobic silica nanoparticles to deliver the enzyme RNase and an anti-phospho Akt antibody intracellularly in MCF7 cells, avoiding endosomal trapping and inducing cell death [96].

Polymeric nanoparticles can mediate the encapsulation of iPeps against TFs in order to modulate gene expression in a non-viral manner. Our group has encapsulated EN1-iPeps targeting the TF EN1 in polymeric poly(glycidyl methacrylate) nanoparticles in conjunction with docetaxel and observed a reduction of in vivo tumor growth in a highly aggressive claudin-low TNBC model [49, 50]. Regarding the nuclear delivery of synthetic TFs, there are many reports in the literature demonstrating proof of concept approaches for effective delivery of synthetic TFs into tumor cells using nanoparticles. Patel et al. developed the NanoScript platform consisting of gold nanoparticles targeted with NLS and encapsulating protein-based synthetic TFs able to increase the expression of a reporter plasmid by over 15-fold in HeLa cells [97]. Similarly, Liu et al. delivered encapsulated artificial TF GAL4-VP16 together with a luciferase reporter plasmid in supramolecular nanoparticles functionalised with RGD and TAT peptides to HeLa cells which showed high expression of the reporter gene [98]. Lastly, a focus of intense interest is the nanovehicle-mediated delivery of reagents for genome engineering in cancer, such as designer zinc finger TFs and CRISPR systems. Our laboratory has successfully delivered CRISPR–dCas9 systemically into MCF7 xenografts using dendritic polymers to reactivate the tumor suppressor genes MASPIN and CCN6 in the tumor site, leading to potent and long-lasting cancer growth inhibition [99].

## Concluding remarks

TFs and other molecules operating through protein–protein interactions are master regulators of oncogenic gene cascades. Due to their high cellular compartmentalization and the featureless nature of protein interfaces, their targeting is challenging. Peptides are a rich source of bioactive compounds that could potentially overcome this problem, providing novel anticancer therapeutics which target difficult-to-inhibit oncogenic drivers. Peptides possess high versatility, specificity, are small, and relatively cheap to synthesize. Their specificity and selectivity are not compromised by de novo point mutations frequently arising in heterogenous tumors as compared to antibodies. Relevant studies have witnessed the potential of iPeps to inhibit central oncogenes such as MYC, HOX, KRAS, BCL-2, and HDM2/HDMX both in vitro and in vivo. Importantly, these studies have facilitated the recent therapeutic application of iPeps against BCL-2 and HDM2/HDMX in the clinical setting for the treatment of a wide range of hematologic and solid malignancies. However, iPep technology will need to overcome certain obstacles to maximize their therapeutic potential. Many areas of improvement include cellular penetrability and half-life in circulation for which a varied range of modifications have been described. Undoubtedly, further improvements and optimizations of current iPeps in development will accelerate their translation to the clinic.

The references marked with an asterix can be found in Supplementary Information.

## Supplementary information

Supplemental Material
