# Supplementary material for: Precision medicine by designer interference peptides: applications in oncology and molecular therapeutics
Source: Oncogene. 2019 Oct 21;39(6):1167–84. doi: 10.1038/s41388-019-1056-3 (PMC7002299; doi:10.1038/s41388-019-1056-3)
Supplement: Supplementary file 1 — Supplemental Material [file 41388_2019_1056_MOESM1_ESM.pdf]

References

Marked with an asterix in the text

101* Abba MC, Laguens RM, Dulout FN, Golijow CD. The c-myc activation in cervical carcinomas and HPV 16 infections. *Mutat Res* 2004; 557: 151-158.

102* Abe M, Hamada J, Takahashi O, Takahashi Y, Tada M, Miyamoto M *et al*. Disordered expression of HOX genes in human non-small cell lung cancer. *Oncol Rep* 2006; 15: 797-802.

103* Alberi L, Sgado P, Simon HH. Engrailed genes are cell-autonomously required to prevent apoptosis in mesencephalic dopaminergic neurons. *Development* 2004; 131: 3229-3236.

104* Alters SE, McLaughlin B, Spink B, Lachinyan T, Wang CW, Podust V *et al*. GLP2-2G-XTEN: a pharmaceutical protein with improved serum half-life and efficacy in a rat Crohn's disease model. *PLoS One* 2012; 7: e50630.

105* Annibali D, Whitfield JR, Favuzzi E, Jauset T, Serrano E, Cuartas I *et al*. Myc inhibition is effective against glioma and reveals a role for Myc in proficient mitosis. *Nat Commun* 2014; 5: 4632.

106* Barrett J, Birrer MJ, Kato GJ, Dosakaakita H, Dang CV. Activation Domains of L-Myc and C-Myc Determine Their Transforming Potencies in Rat Embryo Cells. *Molecular and Cellular Biology* 1992; 12: 3130-3137.

107* Baudino TA, McKay C, Pendeville-Samain H, Nilsson JA, Maclean KH, White EL *et al*. c-Myc is essential for vasculogenesis and angiogenesis during development and tumor progression. *Genes & Development* 2002; 16: 2530-2543.

108* Berg T, Cohen SB, Desharnais J, Sonderegger C, Maslyar DJ, Goldberg J *et al*. Small-molecule antagonists of Myc/Max dimerization inhibit Myc-induced transformation of chicken embryo fibroblasts. *Proc Natl Acad Sci U S A* 2002; 99: 3830-3835.

109* Bister K, Jansen HW. Oncogenes in Retroviruses and Cells - Biochemistry and Molecular-Genetics. *Advances in Cancer Research* 1986; 47: 99-188.

110* Blackwell TK, Kretzner L, Blackwood EM, Eisenman RN, Weintraub H. Sequence-Specific DNA-Binding by the C-Myc Protein. *Science* 1990; 250: 1149-1151.

111* Bouwhuis MG, Suciu S, Testori A, Kruit WH, Sales F, Patel P *et al*. Phase III trial comparing adjuvant treatment with pegylated interferon Alfa-2b versus observation: prognostic significance of autoantibodies--EORTC 18991. *J Clin Oncol* 2010; 28: 2460-2466.

112* Brodeur GM, Seeger RC, Schwab M, Varmus HE, Bishop JM. Amplification of N-myc in untreated human neuroblastomas correlates with advanced disease stage. *Science* 1984; 224: 1121-1124.

113* Chauhan J, Wang H, Yap JL, Sabato PE, Hu A, Prochownik EV *et al*. Discovery of methyl 4'-methyl-5-(7-nitrobenzoc1,2,5oxadiazol-4-yl)-1,1'-biphenyl-3-carboxylate, an improved small-molecule inhibitor of c-Myc-max dimerization. *ChemMedChem* 2014; 9: 2274-2285.

114* Chen H, Zhang H, Lee J, Liang X, Wu X, Zhu T *et al*. HOXA5 acts directly downstream of retinoic acid receptor beta and contributes to retinoic acid-induced apoptosis and growth inhibition. *Cancer Res* 2007; 67: 8007-8013.

115* Clausen DM, Guo J, Parise RA, Beumer JH, Egorin MJ, Lazo JS *et al*. In vitro cytotoxicity and in vivo efficacy, pharmacokinetics, and metabolism of 10074-G5, a novel small-molecule inhibitor of c-Myc/Max dimerization. *J Pharmacol Exp Ther* 2010; 335: 715-727.

116* Costa BM, Smith JS, Chen Y, Chen J, Phillips HS, Aldape KD *et al*. Reversing HOXA9 oncogene activation by PI3K inhibition: epigenetic mechanism and prognostic significance in human glioblastoma. *Cancer Res* 2010; 70: 453-462.

117* Dang CV. c-Myc target genes involved in cell growth, apoptosis, and metabolism. *Mol Cell Biol* 1999; 19: 1-11.

118* Dang CV, O'Donnell KA, Zeller KI, Nguyen T, Osthus RC, Li F. The c-Myc target gene network. *Semin Cancer Biol* 2006; 16: 253-264.

119* Dang CV. MYC on the path to cancer. *Cell* 2012; 149: 22-35.

120* Delgado MD, Leon J. Myc roles in hematopoiesis and leukemia. *Genes Cancer* 2010; 1: 605-616.

121* Drygin D, Siddiqui-Jain A, O'Brien S, Schwaebe M, Lin A, Bliesath J *et al*. Anticancer activity of CX-3543: a direct inhibitor of rRNA biogenesis. *Cancer Res* 2009; 69: 7653-7661.

122* Eklund EA. The role of HOX genes in malignant myeloid disease. *Curr Opin Hematol* 2007; 14: 85-89.

123* Erazo-Oliveras A, Muthukrishnan N, Baker R, Wang TY, Pellois JP. Improving the endosomal escape of cell-penetrating peptides and their cargos: strategies and challenges. *Pharmaceuticals* (Basel) 2012; 5: 1177-1209.

124* Furuse J, Kurata T, Okano N, Fujisaka Y, Naruge D, Shimizu T *et al*. An early clinical trial of Salirasib, an oral RAS inhibitor, in Japanese patients with relapsed/refractory solid tumors. *Cancer Chemother Pharmacol* 2018; 82: 511-519.

125* Garton M, Nim S, Stone TA, Wang KE, Deber CM, Kim PM. Method to generate highly stable D-amino acid analogs of bioactive helical peptides using a mirror image of the entire PDB. *Proc Natl Acad Sci U S A* 2018; 115: 1505-1510.

126* Gebhardt A, Frye M, Herold S, Benitah SA, Braun K, Samans B *et al*. Myc regulates keratinocyte adhesion and differentiation via complex formation with Miz1. *J Cell Biol 2006*; 172: 139-149.

127* Grushko TA, Dignam JJ, Das S, Blackwood AM, Perou CM, Ridderstrale KK *et al*. MYC is amplified in BRCA1-associated breast cancers. *Clinical Cancer Research* 2004; 10: 499-507.

128* Guo J, Parise RA, Joseph E, Egorin MJ, Lazo JS, Prochownik EV *et al*. Efficacy, pharmacokinetics, tisssue distribution, and metabolism of the Myc-Max disruptor, 10058-F4 Z,E-5-4-ethylbenzylidine-2-thioxothiazolidin-4-one, in mice. *Cancer Chemother Pharmacol* 2009; 63: 615-625.

129* Hanahan D, Weinberg RA. The hallmarks of cancer. *Cell* 2000; 100: 57-70.

130* Hart JR, Garner AL, Yu J, Ito Y, Sun M, Ueno L *et al*. Inhibitor of MYC identified in a Krohnke pyridine library. *Proc Natl Acad Sci U S A* 2014; 111: 12556-12561.

131* Hoffmann K, Milech N, Juraja SM, Cunningham PT, Stone SR, Francis RW *et al*. A platform for discovery of functional cell-penetrating peptides for efficient multi-cargo intracellular delivery. *Sci Rep* 2018; 8: 12538.

132* Hong CS, Jeong O, Piao Z, Guo C, Jung MR, Choi C *et al*. HOXB5 induces invasion and migration through direct transcriptional up-regulation of beta-catenin in human gastric carcinoma. *Biochem J* 2015; 472: 393-403.

133* Jin K, Kong X, Shah T, Penet MF, Wildes F, Sgroi DC *et al*. The HOXB7 protein renders breast cancer cells resistant to tamoxifen through activation of the EGFR pathway. *Proc Natl Acad Sci U S A* 2012; 109: 2736-2741.

134* Joliot AH, Triller A, Volovitch M, Pernelle C, Prochiantz A. alpha-2,8-Polysialic acid is the neuronal surface receptor of antennapedia homeobox peptide. *New Biol* 1991; 3: 1121-1134.

135* Jones S, Thornton JM. Principles of protein-protein interactions. *Proc Natl Acad Sci U S A* 1996; 93: 13-20.

136* Kachgal S, Mace KA, Boudreau NJ. The dual roles of homeobox genes in vascularization and wound healing. *Cell Adh Migr* 2012; 6: 457-470.

137* Kim YJ, Yoon HY, Kim JS, Kang HW, Min BD, Kim SK *et al*. HOXA9, ISL1 and ALDH1A3 methylation patterns as prognostic markers for nonmuscle invasive bladder cancer: array-based DNA methylation and expression profiling. *Int J Cancer* 2013; 133: 1135-1142.

138* Kim YJ, Sung M, Oh E, Vrancken MV, Song JY, Jung K *et al*. Engrailed 1 overexpression as a potential prognostic marker in quintuple-negative breast cancer. *Cancer Biol Ther* 2018; 19: 335-345.

139* Lebert-Ghali CE, Fournier M, Dickson GJ, Thompson A, Sauvageau G, Bijl JJ. HoxA cluster is haploinsufficient for activity of hematopoietic stem and progenitor cells. *Exp Hematol* 2010; 38: 1074-1086 e1071-1075.

140* Liao WT, Jiang D, Yuan J, Cui YM, Shi XW, Chen CM *et al*. HOXB7 as a prognostic factor and mediator of colorectal cancer progression. *Clin Cancer Res* 2011; 17: 3569-3578.

141* Little CD, Nau MM, Carney DN, Gazdar AF, Minna JD. Amplification and expression of the c-myc oncogene in human lung cancer cell lines. *Nature* 1983; 306: 194-196.

142* Mateyak MK, Obaya AJ, Sedivy JM. c-Myc regulates cyclin D-Cdk4 and-Cdk6 activity but affects cell cycle progression at multiple independent points. *Mol Cell Biol* 1999; 19: 4672-4683.

143* McGinnis W, Hart CP, Gehring WJ, Ruddle FH. Molecular cloning and chromosome mapping of a mouse DNA sequence homologous to homeotic genes of Drosophila. *Cell* 1984; 38: 675-680.

144* Milech N, Longville BA, Cunningham PT, Scobie MN, Bogdawa HM, Winslow S *et al*. GFP-complementation assay to detect functional CPP and protein delivery into living cells. *Sci Rep* 2015; 5: 18329.

145* Miller GJ, Miller HL, van Bokhoven A, Lambert JR, Werahera PN, Schirripa O *et al*. Aberrant HOXC expression accompanies the malignant phenotype in human prostate. *Cancer Res* 2003; 63: 5879-5888.

146* Morgan R, Bryan RT, Javed S, Launchbury F, Zeegers MP, Cheng KK *et al*. Expression of Engrailed-2 (EN2) protein in bladder cancer and its potential utility as a urinary diagnostic biomarker. *Eur J Cancer* 2013; 49: 2214-2222.

147* Morgan R, El-Tanani M, Hunter KD, Harrington KJ, Pandha HS. Targeting HOX/PBX dimers in cancer. *Oncotarget* 2017; 8: 32322-32331.

148* Nau MM, Brooks BJ, Battey J, Sausville E, Gazdar AF, Kirsch IR *et al*. L-myc, a new myc-related gene amplified and expressed in human small cell lung cancer. *Nature* 1985; 318: 69-73.

149* Nesbit CE, Grove LE, Yin X, Prochownik EV. Differential apoptotic behaviors of c-myc, N-myc, and L-myc oncoproteins. *Cell Growth Differ* 1998; 9: 731-741.

150* Ou TM, Lu YJ, Zhang C, Huang ZS, Wang XD, Tan JH *et al*. Stabilization of G-quadruplex DNA and down-regulation of oncogene c-myc by quindoline derivatives. *J Med Chem* 2007; 50: 1465-1474.

151* Ozpolat B, Sood AK, Lopez-Berestein G. Nanomedicine based approaches for the delivery of siRNA in cancer. *J Intern Med* 2010; 267: 44-53.

152* Papadopoulos DK, Vukojevic V, Adachi Y, Terenius L, Rigler R, Gehring WJ. Function and specificity of synthetic Hox transcription factors in vivo. *Proc Natl Acad Sci U S A* 2010; 107: 4087-4092.

153* Platais C, Radhakrishnan R, Niklander Ebensperger S, Morgan R, Lambert DW, Hunter KD. Targeting HOX-PBX interactions causes death in oral potentially malignant and squamous carcinoma cells but not normal oral keratinocytes. *BMC Cancer* 2018; 18: 723.

154* Prendergast GC, Ziff EB. Methylation-sensitive sequence-specific DNA binding by the c-Myc basic region. *Science* 1991; 251: 186-189.

155* Qvit N, Rubin SJS, Urban TJ, Mochly-Rosen D, Gross ER. Peptidomimetic therapeutics: scientific approaches and opportunities. *Drug Discov Today* 2017; 22: 454-462.

156* Roberts AW, Seymour JF, Brown JR, Wierda WG, Kipps TJ, Khaw SL *et al.* Substantial susceptibility of chronic lymphocytic leukemia to BCL2 inhibition: results of a phase I study of navitoclax in patients with relapsed or refractory disease. *J Clin Oncol* 2012; 30: 488-496.

157* Savino M, Annibali D, Carucci N, Favuzzi E, Cole MD, Evan GI *et al.* The Action Mechanism of the Myc Inhibitor Termed Omomyc May Give Clues on How to Target Myc for Cancer Therapy. *Plos One* 2011; 6.

158* Scott MP, Weiner AJ. Structural relationships among genes that control development: sequence homology between the Antennapedia, Ultrabithorax, and fushi tarazu loci of Drosophila. *Proc Natl Acad Sci U S A* 1984; 81: 4115-4119.

159* Sgado P, Alberi L, Gherbassi D, Galasso SL, Ramakers GM, Alavian KN *et al.* Slow progressive degeneration of nigral dopaminergic neurons in postnatal Engrailed mutant mice. *Proc Natl Acad Sci U S A* 2006; 103: 15242-15247.

160* Sheiness D, Bishop JM. DNA and RNA from uninfected vertebrate cells contain nucleotide sequences related to the putative transforming gene of avian myelocytomatosis virus. *J Virol* 1979; 31: 514-521.

161* Shi Y, Glynn JM, Guilbert LJ, Cotter TG, Bissonnette RP, Green DR. Role for c-myc in activation-induced apoptotic cell death in T cell hybridomas. *Science* 1992; 257: 212-214.

162* Shustov AR, Horwitz SM, Zain J, Patel MR, Goel S, Sokol M *et al*. Preliminary Results of the Stapled Peptide ALRN-6924, a Dual Inhibitor of MDMX and MDM2, in Two Phase IIa Dose Expansion Cohorts in Relapsed/Refractory TP53 Wild-Type Peripheral T-Cell Lymphoma. *Blood* 2018: 1623.

163* Sikora K, Chan S, Evan G, Gabra H, Markham N, Stewart J *et al*. c-myc oncogene expression in colorectal cancer. *Cancer* 1987; 59: 1289-1295.

164* Soucek L, Whitfield J, Martins CP, Finch AJ, Murphy DJ, Sodir NM *et al*. Modelling Myc inhibition as a cancer therapy. *Nature* 2008; 455: 679-683.

165* Soucek L, Whitfield JR, Sodir NM, Masso-Valles D, Serrano E, Karnezis AN *et al*. Inhibition of Myc family proteins eradicates KRas-driven lung cancer in mice. *Genes Dev* 2013; 27: 504-513.

166* Stellas D, Szabolcs M, Koul S, Li Z, Polyzos A, Anagnostopoulos C *et al*. Therapeutic effects of an anti-Myc drug on mouse pancreatic cancer. *J Natl Cancer Inst* 2014; 106.

167* Strieder V, Lutz W. Regulation of N-myc expression in development and disease. *Cancer Letters* 2002; 180: 107-119.

168* Takahashi K, Yamanaka S. Induction of pluripotent stem cells from mouse embryonic and adult fibroblast cultures by defined factors. *Cell* 2006; 126: 663-676.

169* Taylor HS. Transcriptional regulation of implantation by HOX genes. *Rev Endocr Metab Disord* 2002; 3: 127-132.

170* Teo WW, Merino VF, Cho S, Korangath P, Liang X, Wu RC *et al*. HOXA5 determines cell fate transition and impedes tumor initiation and progression in breast cancer through regulation of E-cadherin and CD24. *Oncogene* 2016; 35: 5539-5551.

171* Tolcher AW, Papadopoulos KP, Patnaik A, Rasco DW, Martinez D, Wood DL *et al*. Safety and activity of DCR-MYC, a first-in-class Dicer-substrate small interfering RNA (DsiRNA) targeting MYC, in a phase I study in patients with advanced solid tumors. *Journal of Clinical Oncology* 2015; 33.

172* Veine DM, Yao H, Stafford DR, Fay KS, Livant DL. A D-amino acid containing peptide as a potent, noncovalent inhibitor of alpha5beta1 integrin in human prostate cancer invasion and lung colonization. *Clin Exp Metastasis* 2014; 31: 379-393.

173* Wang HB, Hammoudeh DI, Follis AV, Reese BE, Lazo JS, Metallo SJ *et al*. Improved low molecular weight Myc-Max inhibitors. *Molecular Cancer Therapeutics 2007*; 6: 2399-2408.

174* Wang J, Hu X, Xiang D. Nanoparticle drug delivery systems: an excellent carrier for tumor peptide vaccines. *Drug Deliv* 2018; 25: 1319-1327.

175* Wang JL, Wang H, Li ZZ, Wu QL, Lathia JD, McLendon RE *et al*. c-Myc Is Required for Maintenance of Glioma Cancer Stem Cells. *Plos One* 2008; 3.

176* Watt PM. Screening for peptide drugs from the natural repertoire of biodiverse protein folds. Nat Biotechnol 2006; 24: 177-183.

177* Wender PA, Mitchell DJ, Pattabiraman K, Pelkey ET, Steinman L, Rothbard JB. The design, synthesis, and evaluation of molecules that enable or enhance cellular uptake: peptoid molecular transporters. *Proc Natl Acad Sci U S A* 2000; 97: 13003-13008.

178* Xu J, Chen Y, Olopade OI. MYC and Breast Cancer. *Genes Cancer* 2010; 1: 629-640.

179* Yamashita T, Tazawa S, Yawei Z, Katayama H, Kato Y, Nishiwaki K *et al.* Suppression of invasive characteristics by antisense introduction of overexpressed HOX genes in ovarian cancer cells. *Int J Oncol* 2006; 28: 931-938.

180* Yan M, Du J, Gu Z, Liang M, Hu Y, Zhang W *et al.* A novel intracellular protein delivery platform based on single-protein nanocapsules. *Nat Nanotechnol* 2010; 5: 48-53.

181* Yin X, Giap C, Lazo JS, Prochownik EV. Low molecular weight inhibitors of Myc-Max interaction and function. *Oncogene* 2003; 22: 6151-6159.

182* Druker BJ, Sawyers CL, Kantarjian H, Resta DJ, Reese SF, Ford JM *et al.* Activity of a specific inhibitor of the BCR-ABL tyrosine kinase in the blast crisis of chronic myeloid leukemia and acute lymphoblastic leukemia with the Philadelphia chromosome. *N Engl J Med* 2001; 344: 1038-42.

183* Vagner J, Qu H, Hruby VJ. Peptidomimetics, a synthetic tool of drug discovery. *Curr Opin Chem Biol* 2008; 12: 292-296.

184* Fletcher S, Prochownik EV. Small-molecule inhibitors of the Myc oncoprotein. *Biochim Biophys Acta* 2015; 1849: 525-543.

185* Shen LY, Zhou T, Du YB, Shi Q, Chen KN. Targeting HOX/PBX dimer formation as a potential therapeutic option in esophageal squamous cell carcinoma. *Cancer Sci* 2019; 110:1735-1745.

186* Ando H, Natsume A, Senga T, Watanabe R, Ito I, Ohno M *et al*. Peptide-based inhibition of the HOXA9/PBX interaction retards the growth of human meningioma. *Cancer Chemother Pharmacol* 2014; 73: 53-60.

187* Errico MC, Felicetti F, Bottero L, Mattia G, Boe A, Felli N *et al*. The abrogation of the HOXB7/PBX2 complex induces apoptosis in melanoma through the miR-221&222-c-FOS pathway. *Int J Cancer* 2013; 133: 879-892.

188* Lee EF, Czabotar PE, Yang H, Sleebs BE, Lessene G, Colman PM *et al*. Conformational changes in Bcl-2 pro-survival proteins determine their capacity to bind ligands. *J Biol Chem* 2009; 284: 30508-30517.

189* Ji Y, Majumder S, Millard M, Borra R, Bi T, Elnagar AY *et al*. In vivo activation of the p53 tumor suppressor pathway by an engineered cyclotide. *J Am Chem Soc* 2013; 135: 11623-11633.

190* Arranz-Gibert P, Ciudad S, Seco J, García J, Giralt E, Teixidó M. Immunosilencing peptides by stereochemical inversion and sequence reversal: retro-D-peptides. *Sci Rep* 2018; 8: 6446.

191* Dharap SS, Qiu B, Williams GC, Sinko P, Stein S, Minko T. Molecular targeting of drug delivery systems to ovarian cancer by BH3 and LHRH peptides. *J Control Release* 2003;91: 61-73.

192* Guharoy M, Chakrabarti P. Secondary structure based analysis and classification of biological interfaces: identification of binding motifs in protein-protein interactions. *Bioinformatics* 2007;23: 1909-1918.

193* Tan YS, Lane DP, Verma CS. Stapled peptide design: principles and roles of computation. *Drug Discov Today* 2016; 21: 1642-1653.

194* Grossmann TN, Yeh JT, Bowman BR, Chu Q, Moellering RE, Verdine GL. Inhibition of oncogenic Wnt signaling through direct targeting of β-catenin. *Proc Natl Acad Sci U S A* 2012; 109: 17942-17947.

195* Moellering RE, Cornejo M, Davis TN, Del Bianco C, Aster JC, Blacklow SC *et al*. Direct inhibition of the NOTCH transcription factor complex. *Nature* 2009; 462: 182-188.

196* Jawa V, Cousens LP, Awwad M, Wakshull E, Kropshofer H, De Groot AS. T-cell dependent immunogenicity of protein therapeutics: Preclinical assessment and mitigation. *Clin Immunol* 2013; 149: 534-455.

197* Harris JM, Chess RB. Effect of pegylation on pharmaceuticals. *Nat Rev Drug Discov* 2003; 2: 214-221.
